# CURRENT STATUS OF THE FUKUSHIMA HEALTH MANAGEMENT SURVEY

**DOI:** 10.1093/rpd/ncy138

**Published:** 2018-08-31

**Authors:** Atsushi Kumagai, Koichi Tanigawa

**Affiliations:** 1 Education Center for Disaster Medicine, Fukushima Medical University, 1 Hikariga-oka, Fukushima, Japan; 2 Fukushima Global Medical Science Center, Fukushima Medical University, 1 Hikariga-oka, Fukushima, Japan

## Abstract

The Fukushima Health Management Survey (FHMS) was implemented in the wake of the 2011 Fukushima Daiichi Nuclear Power Plant accident. The primary purpose of this survey was to monitor the long-term health of residents, promote their future well-being and confirm whether long-term low-dose radiation exposure affects health. The FHMS results indicated very low-radiation exposure doses among residents and that no discernible increased incidence of radiation-related health effects could be expected. However, psychological distress was found to be far greater among people in Fukushima than those in other areas affected by the accident’s preceding Great East Japan Earthquake and the resultant tsunami. Additionally, prevalence of lifestyle-related health problems such as being overweight, hypertension, diabetes mellitus, dyslipidaemia and liver dysfunction increased among evacuees. Thyroid examinations of asymptomatic individuals, using ultrasound techniques, also contributed to public concern and fear about the health effects of radiation. The FHMS ultimately revealed that ethical considerations are important in the design and implementation of health surveillance and epidemiological studies.

After the Fukushima Daiichi Nuclear Power Plant Accident in 2011, 160 000 people from the plant’s vicinity were evacuated either mandatorily or voluntarily. A great deal of uncertainty and concerns were present about the post-accident health effects of radiation. In that challenging climate, the Fukushima prefectural government decided to launch the Fukushima Health Management Survey (FHMS), and Fukushima Medical University was entrusted with its design and implementation^(1)^. The survey’s primary aims were to monitor the long-term health of residents, promote their well-being and determine whether long-term, low-dose radiation exposure has any effects of health. The survey had two major components: Basic and Detailed Surveys (Figure 1). The Basic Survey estimated external radiation exposure of 2 million Fukushima residents and visitors to Fukushima as of 11 March 2011. There were four Detailed Surveys; Thyroid Ultrasound Examination (subjects: 360 000 children aged ≤18 years), Comprehensive Health Check (subjects: 210 000 former residents of the evacuation areas), Mental Health and Lifestyle Survey (subjects: 210 000 residents of the evacuation areas) and Pregnancy and Birth Survey (subjects: 16 000 pregnant women in Fukushima).

**Figure 1.**
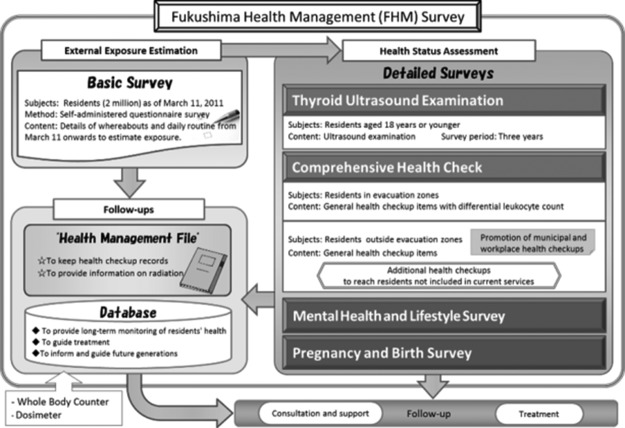
Framework of the FHMS (excerpted from^(1)^).

## BASIC SURVEY

Personal behaviour data were obtained via a questionnaire in order to estimate radiation exposure doses. Doses for the first 4 months after the accident were estimated by superimposing the residents’ behaviour data over γ-ray dose rate maps^(2)^. This survey had estimated individual doses for about 550 000 people as of the end of December 2015. Although the response rate to the Basic Survey was around 27%, the respondents could be considered as representative of all Fukushima residents^(3)^. Table 1 shows the highest exposure dose was 66 mSv, which was observed in a resident who was involved in the emergency operations at the plant. Otherwise, 25 mSv was the highest recorded dose among the Fukushima residents, and the mean dose was 0.8 mSv. Higher doses were observed in the Soso region, which included evacuation zones.

**Table 1.** Estimated external radiation doses (excerpted from^(20)^) as of 31 December 2015.

Effective dose (mSv)	Total	Excluding radiation workers				By area (excluding radiation workers)													
						Kempoku^a^		Kenchu		Kennan		Aizu		Minami-aizu		Soso^b^		Iwaki	
<1	291 093	285 418	62.1%	93.8%	99.8%	24 853	20.1%	57 643	51.5%	25 460	88.2%	44 456	99.3%	4837	99.3%	55 661	77.3%	72 508	99.1%
1–2	148 178	145 845	31.7%			83 056	67.0%	45 780	40.9%	3 386	11.7%	300	0.7%	34	0.7%	12 658	17.6%	631	0.9%
2–3	25 769	25 396	5.5%	5.8%		15 499	12.5%	8 138	7.3%	17	0.1%	25	0.1%	0	—	1687	2.3%	30	0.0%
3–4	1571	1491	0.3%			468	0.4%	423	0.4%	0	—	1	0.0%	0	—	595	0.8%	4	0.0%
4–5	550	504	0.1%	0.2%		40	0.0%	5	0.0%	0	—	0	—	0	—	458	0.6%	1	0.0%
5–6	441	389	0.1%		0.2%	19	0.0%	3	0.0%	0	—	0	—	0	—	366	0.5%	1	0.0%
6–7	268	230	0.1%	0.1%		10	0.0%	1	0.0%	0	—	1	0.0%	0	—	218	0.3%	0	—
7–8	155	116	0.0%			1	0.0%	0	—	0	—	0	—	0	—	115	0.2%	0	—
8–9	118	78	0.0%	0.0%		1	0.0%	0	—	0	—	0	—	0	—	77	0.1%	0	—
9–10	72	41	0.0%			0	—	0	—	0	—	0	—	0	—	41	0.1%	0	—
10–11	69	36	0.0%	0.0%	0.0%	0	—	0	—	0	—	0	—	0	—	36	0.1%	0	—
11–12	52	30	0.0%			1	0.0%	0	—	0	—	0	—	0	—	29	0.0%	0	—
12–13	37	13	0.0%	0.0%		0	—	0	—	0	—	0	—	0	—	13	0.0%	0	—
13–14	34	12	0.0%			0	—	0	—	0	—	0	—	0	—	12	0.0%	0	—
14–15	27	6	0.0%	0.0%		0	—	0	—	0	—	0	—	0	—	6	0.0%	0	—
≥15	314	15	0.0%		0.0%	0	—	0	—	0	—	0	—	0	—	15	0.0%	0	—
Total	468 748	459 620	100.0%	100.0%	100.0%	123 948	100%	111 993	100%	28 863	100%	44 783	100%	4871	100%	71 987	100%	73 175	100%
Max	66 mSv	25 mSv				11 mSv		6.3 mSv		2.6 mSv		6.0 mSv		1.9 mSv		25 mSv		5.9 mSv	
Mean Value	0.9 mSv	0.8 mSv				1.4 mSv		1.0 mSv		0.6 mSv		0.2 mSv		0.1 mSv		0.8 mSv		0.3 mSv	

^a^Including Yamakiya of Kawamata.

^b^Including Namie and Iitate.

Percentage have been rounded and may not total to 100%.

Excluding those with estimation period less than 4 months.

Table 2 shows the first-year doses estimated by the United Nations Scientific Committee on the Effects of Atomic Radiation (UNSCEAR), and the results from the Basic Survey in five municipalities where personal dosemeter results were available^(2, 4–6)^. The effective dose due to external radiation for the first year was estimated by adding the estimated dose from the Basic Survey to individual doses obtained from personal dosemeters. Dose ranges (1.2–2.5 mSv per year) were lower than those in the UNSCEAR report. The major reasons for the differences were dose estimation based on personal behaviour data, use of direct measurement results and effects of decontamination of certain areas.

**Table 2.** Estimated external radiation doses (excerpted from^(20)^).

Municipalities	Basic Survey (First 4 months, regional average)	Personal dosemeter measurement		First-year (Basic Survey + personal dosemeter)	First-year external dose (UNSCEAR)			Lifetime external dose (adults) by UNSCEAR
		Average	Measurement period		Adults	10-year-old children	1-year-old infants	
Fukushima	1.4	0.26	1 September–30 November 2011	2.1	3.02	4.25	5.03	10
Date	1.4	0.17–0.71^a^	1 September–30 November 2011	1.9–3.3	1.95	2.75	3.25	6.5
Nihonmatsu	1.4	0.37–0.41^b^	1 September–30 November 2011	2.4–2.5	2.44	3.43	4.05	8.4
Tamura	1.0	0.10–0.17^c^	30 September 2011–10 January 2012	1.2–1.4	0.52	0.74	0.87	1.6
Koriyama	1.0	0.17	7 November 2011–9 January 2012	1.7	2.01	2.83	3.35	7.0

The first-year doses estimated by UNSCEAR and its comparison with doses extrapolated from the Basic Survey.

UNSCEAR, United Nations Scientific Committee on the Effects of Atomic Radiation.

^a^Averages for five districts ranged from 0.17 to 0.71 mSv.

^b^Averages for elementary school, junior high school and high school children ranged from 0.37 to 0.41 mSv.

^c^Averages for five districts ranged from 0.10 to 0.17 mSv.

Regarding internal exposure, the UNSCEAR report stated that direct measurements of the radioactive content of the whole-body dose due to internal exposure showed lower doses than those estimated by the committee, by a factor of up to around 10.5. The other report, analysing data of caesium content in the body for 174 residents, indicated the mean committed effective dose was far lower in the subjects, even among evacuees from the most highly contaminated areas, such as Namie Town and Iitate Village, than in the UNSCEAR estimation for non-evacuated areas. The 90th percentile committed effective dose for these subjects was around 0.1 mSv and the maximum was 0.63 mSv for one older man^(6)^.

Table 3 shows thyroid exposure doses estimated by the UNSCEAR and the National Institute of Radiological Sciences (NIRS)^(7)^. The NIRS used three approaches to estimate thyroid doses (atmospheric dispersion simulation, whole-body counting data at an early stage and direct measurement of the thyroid) and reported results that were generally lower than district-average doses for the same ages as those estimated in the UNSCEAR report, even when adding in external doses. Dose estimation via ingestion may be the main reason for the difference between the two estimations for people of the evacuated areas.

**Table 3.** Excerpted from^(2)^.

Municipalities (districts)	UNSCEAR (district average, including external dose) (mSv)	NIRS (90th percentile, inhalation dose only) (mSv)	Basic Survey (90th percentile, 4-month external dose only) (mSv)
Iitate	56	30	7–8
Futaba	15–19	30	1–2
Namie	81–83	20	1–2
Kawamata (Yamakiya)	65	10	1–2^a^
Minami Soma	47	20	1–2
Katsurao	73	20	1–2
Hirono	34	20	0–1
Naraha	69–82	10	0–1
Okuma	36	20	1–2
Tomioka	47	10	1–2
Iwaki	52^b^	30	0–1
Non-evacuated areas	33–49^c^	<10	0–1

NIRS, National Institute of Radiological Sciences.

^a^All of Kawamata Town.

^b^Inhalation, 18 mSv; ingestion, 33 mSv; external, 1 mSv.

^c^0–11 mSv due to inhalation.

No discernible increased incidence of radiation-related health effects would be expected based on the reports on estimated effective dose. However, more accurate dose estimations taking into account the effects of behaviour patterns, and other pertinent information, still need to be supplied.

## THYROID ULTRASOUND EXAMINATIONS

**Figure 2.**
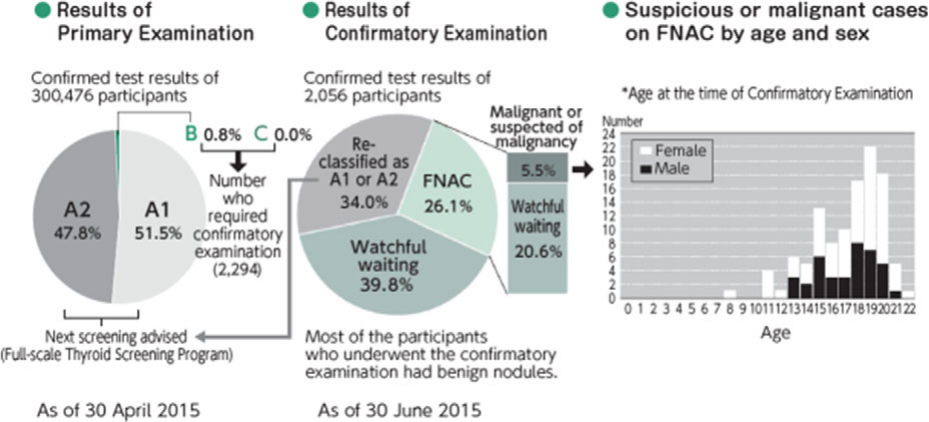
Results of Thyroid Ultrasound Examination (excerpted from^(20)^). A1: no findings; A2: nodules ≤5.0 mm or cysts ≤20 mm; B: nodules ≥5.1 mm or cysts ≥20.1 mm; C: requires immediate examination. FNAC, fine needle aspiration cytology.

Over the last 7 years, thyroid ultrasound examinations using sophisticated technology have been provided to approximately 368 000 Fukushima children aged ≤18 years at the time of the accident. As of 30 April 2015, 300 473 (81.7%) of the children participated in the survey, of which 47.8% had nodules ≤5.0 mm or cysts ≤20 mm, while 2 294 (0.8%) needed confirmatory examinations. Among them, 116 (0.038%) had nodules classified as suspicious or malignant (Figure 2).

Demographic patterns of the suspicious or malignant cases, in that the peak prevalence was 19 years old (at the time of the confirmatory examination), differ from those found after the Chernobyl accident. The UNSCEAR Report 2016 noted that the likelihood of a large number of radiation-induced thyroid cancers in Fukushima Prefecture, such as occurred after the Chernobyl accident, could be discounted because absorbed doses to the thyroid after the Fukushima accident were substantially lower^(7)^. The sensitive ultrasound thyroid screening was expected to detect many thyroid cysts and nodules, including thyroid cancers not normally detectable without systematic screening.

However, the high detection rate of thyroid tumours caused a great deal of concern among children and parents^(8)^. Contrary to the accumulated evidence about low probability of cancer development in the future in Fukushima, people became more concerned, particularly about thyroid cancer.

The goal of screening programs should be to detect disease as early as possible, under the assumption early diagnosis will lead to reduced morbidity and mortality. However, challenges lay in identifying at-risk populations, in which screening yields more benefits than potential harm. There may, for instance, be a situation in which intervention is not fully justified, is associated with psychological stress, or there are ethical considerations such as overdiagnosis, stigmatisation and social impact^(8)^. However, there are no internationally agreed upon criteria for selecting subjects of long-term follow-up; no clear standard protocol exists for such follow-up programs. This is a gap that should be addressed in Fukushima.

## COMPREHENSIVE HEALTH CHECK

Japan has an annual health check-up system implemented among all people aged ≥40 years. Additionally, before children reach age 6, municipalities conduct health check-ups on them at 18 months, 3 years and prior to their entering preschool. School children also receive basic annual health check-ups. In the Comprehensive Heath Check of the FHMS, additional blood tests were given in these health check-ups. Table 4 shows increased prevalence of obesity, glucose intolerance, liver dysfunction and hypertension observed among subjects after the accident.

**Table 4.** Proportions of obesity, glucose intolerance, liver dysfunction and hypertension before and after the Fukushima accident (excerpted from^(20)^).

	Obesity		Impaired glucose intolerance (HbA1c ≥ 16.5%)		Hepatic dysfunction (ALT of ≥51 U/L)		Hypertension (diastolic pressure of ≥90 mmHg)	
	Male (%)	Female (%)	Male (%)	Female (%)	Male (%)	Female (%)	Male (%)	Female (%)
FY 2008	30	31	4.1	2.9	4.3	1.8	16.4	11.6
FY 2009	30	30	4.5	2.8	4.0	1.8	15.4	9.6
FY 2010	30	28	4.4	2.7	3.8	1.7	15.7	10.3
FY 2011	42	34	7.0	3.4	11.0	4.4	19.7	11.6
FY 2012	38	33	5.1	2.7	7.7	3.9	15.8	10.1

**Figure 3.**
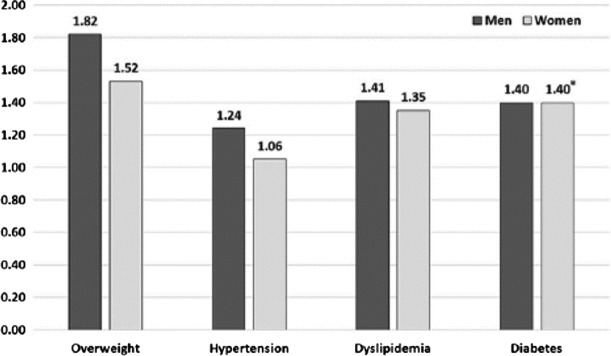
Changes in proportion of overweight/obese people before and after the Great East Japan Earthquake, stratified by sex and evacuation status (excerpted from^(9)^).

**Figure 4.**
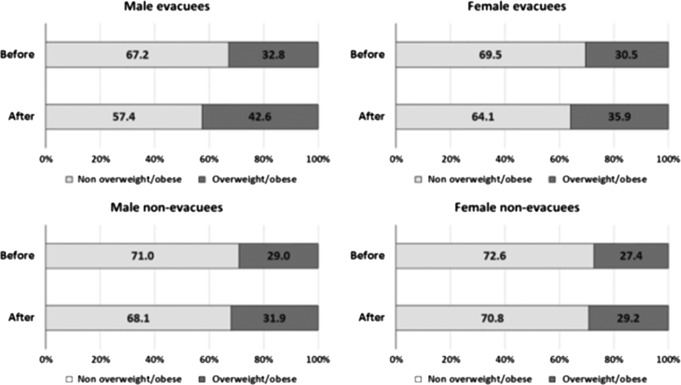
Multivariable-adjusted hazard ratios of overweight/obese, hypertension, dyslipidaemia and diabetes mellitus for evacuation (excerpted from^(9)^).

Ohira *et al.* performed more detailed analyses about the accident’s effects on health^(9)^. They analysed data of 27 486 residents who lived near the Fukushima Daiichi Nuclear Power Plant before the accident and who received follow-up examinations after the accident. The average follow-up period was 1.6 years, and the residents were divided into two groups for analysis: evacuees (*n* = 9671) and non-evacuees (*n* = 17 815). The proportion of overweight/obese people in both groups significantly increased after the accident, although the changes in proportions were greater among evacuees than non-evacuees (*p* < 0.001) (Figures 3 and 4). Evacuation was associated with an increased risk of becoming overweight/obese, and the hazard ratio of evacuation was 1.82 for men and 1.52 for women. Increased hazard ratios for hypertension, dyslipidaemia and diabetes were also found associated with evacuation.

The prevalence of atrial fibrillation increased significantly among residents after the accident (before 1.9% vs. after 2.4%; *p* < 0.001)^(10)^. Prevalence rates for polycythaemia also increased among the evacuees after the accident^(9)^. The findings of this longitudinal study—i.e. increases in body weight, increased proportions of high blood pressure, diabetes, dyslipidaemia, liver dysfunction, atrial fibrillation and polycythaemia—suggested evacuation may trigger greater prevalence of cardiovascular diseases unless these associated health problems are properly addressed.

## MENTAL HEALTH AND LIFESTYLE SURVEY

This survey’s items included mental status, physical status, activities during the preceding 6 months, perception of radiation risk, experience during the earthquake and time of relocation. Self-administered questionnaires (Kessler six-item questionnaire [K6], Posttraumatic Stress Disorder Checklist Stressor-Specific Version [PCL-S]) were used to evaluate the mental status of evacuees aged ≥16 years. Parents were asked to evaluate the behaviour of their children using the Strengths and Difficulties Questionnaire (SDQ).

The proportion of those with psychological distress, including children, was far greater in this survey (14.6%) than that found in other areas affected by the earthquake and tsunami (6.2%) or the Japanese population under normal circumstances (4.2–4.4%)^(11–13)^.

**Figure 5.**
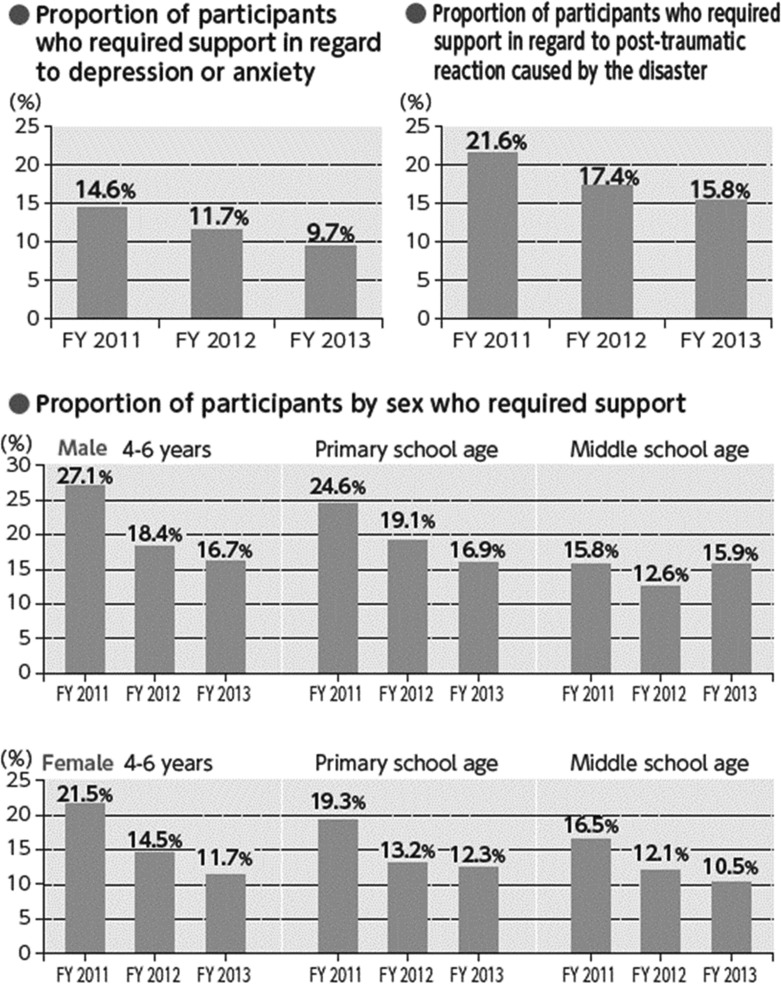
Psychological effects of the Fukushima accident (excerpted from^(20)^).

The proportion of residents who required support for depressive symptoms and anxiety has been decreasing gradually but remains at higher levels than in the general population (Figure 5).

**Figure 6.**
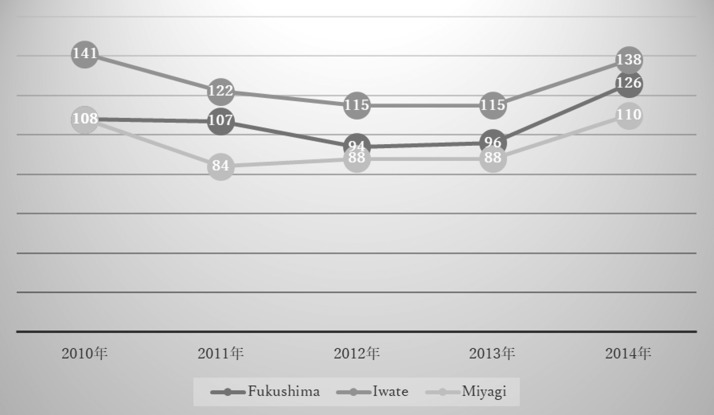
Standardised suicide mortality ratio in the aftermath of the Great East Japan Earthquake^(14)^.

The biggest concern surrounding mental problems is suicide. The Tohoku region of north-eastern Japan is notorious for its high suicide rate. Iwate and Miyagi were prefectures in Tohoku severely damaged by the earthquake and tsunami. Soon after the events, the suicide rate decreased temporarily in the two prefectures and then increased gradually (Figure 6). However, in Fukushima, the suicide rate did not drop as much as in the other two prefectures and, in comparison, is increasing more rapidly^(14)^.

## PREGNANCY AND BIRTH SURVEY

Disasters have particularly harmful effects on vulnerable populations, such as pregnant women. After nuclear accidents in particular, pregnant women fear for their expected children’s and their own health. Therefore, while the survey was taking place, telephone counselling or email support was provided^(15)^. Despite the trying climate after the compound disaster of the earthquake, tsunami and severe nuclear power plant accident, we did not observe any increase in abnormal deliveries, nor did we see deterioration of maternal health (Table 5). However, telephone support was provided for over 1000 mothers per year. Soon after the accident, radiation was their major concern, but over time the focus shifted to mental/psychological health and general health.

**Table 5.** Results of Pregnancy and Birth Survey (excerted from^(20)^).

	Rate of preterm deliveries	Rate of low-birth weight infants	Rate of congenital anomalies	
FY 2011	4.75 (5.7)	8.9 (9.6)	2.85	(3~5)^a^
FY 2012	5.74 (5.7)	9.6 (9.6)	2.39	
FY 2013	5.40 (5.8)	9.9 (9.6)	2.35	
FY 2014	5.43 (5.7)	10.1 (9.5)	2.30	

Figures in the brackets are the proportion of preterm deliveries and incidence of low-birth weight infants reported in the Vital Statistics conducted by the Ministry of Health, Labour and Welfare for the same fiscal year.

^a^Figures in the brackets are the generally reported incidence of congenital anomalies.

**Figure 7.**
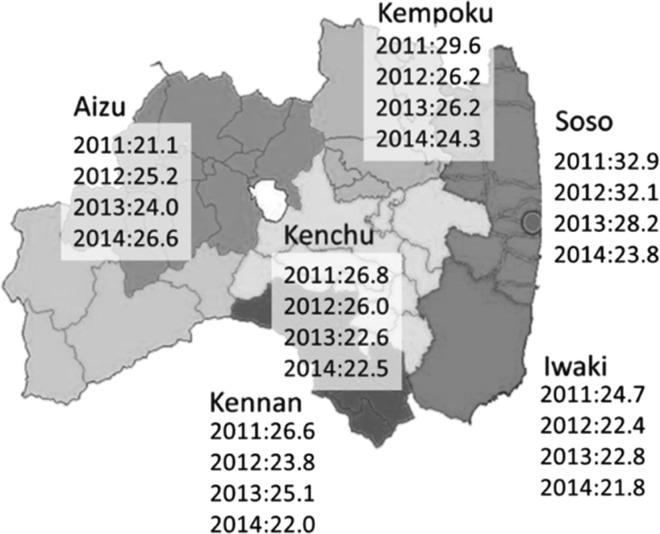
Regional variation in frequency (%) of mothers diagnosed as positive for depressive symptoms^(16)^.

One-fourth of mothers surveyed in 2011 showed depressive symptoms, with the highest proportion observed in the Soso region, where the Fukushima Nuclear Power Plant is located (Figure 7)^(16)^. Although a gradual decline was seen in the proportion of such mothers, 20% in 2014 were diagnosed as positive for depression. The proportion of mothers assessed as needing telephone counselling remained at about 15% during the first 3 years and decreased slightly thereafter.

## LESSONS LEARNED FROM THE FUKUSHIMA ACCIDENT

The FHMS revealed adverse effects on the mental health of residents, including children and pregnant women, various health problems caused by long-term relocation and ethical issues related to thyroid ultrasound examinations that were more obvious than the direct health risks of radiation^(17)^. These findings indicate it is essential to balance the risks of radiation with other health effects after an accident and to develop specific measures to mitigate overall health risks.

Before the Fukushima accident, very few scientists had paid considerable attention to the importance of planning for health surveys following a nuclear power plant accident^(18)^. Despite severe difficulties after the compound disaster in Fukushima, the basic concept of the FHMS, which included not only the health effects of radiation but also other health issues, such as mental health and health consequences of the long-term relocation, was developed, and the survey was implemented within a short period of time. However, we have had to face ethical issues related to this surveillance, particularly in the thyroid ultrasound examinations.

Nuclear Emergency Situations—Improvement of Medical And Health Surveillance (SHAMISEN), a project funded by the Open Project For European Radiation Research Area, aimed to develop recommendations for medical and health surveillance of populations affected by previous and future radiation accidents based on lessons learned from the past accidents, including the Fukushima accident^(19)^. The SHAMISEN recommendations state that ‘Management of radiological accidents also raises important ethical issues. Although the majority of radiation protection actions, including health surveillance, are directed towards reducing the impacts of exposure to ionising radiation, most of these carry with them a multitude of direct and indirect consequences that can have a large impact on the welfare of affected populations. Ethical considerations are also important for the design and implementation of health surveillance and epidemiological studies’.
